# Pollination by the locally endangered island flying fox (*Pteropus hypomelanus*) enhances fruit production of the economically important durian (*Durio zibethinus*)

**DOI:** 10.1002/ece3.3213

**Published:** 2017-09-18

**Authors:** Sheema A. Aziz, Gopalasamy R. Clements, Kim R. McConkey, Tuanjit Sritongchuay, Saifful Pathil, Muhammad Nur Hafizi Abu Yazid, Ahimsa Campos‐Arceiz, Pierre‐Michel Forget, Sara Bumrungsri

**Affiliations:** ^1^ Rimba Kuala Lumpur Malaysia; ^2^ Département Adaptations du Vivant UMR MECADEV 7179 CNRS‐MNHN Muséum National d'Histoire Naturelle Brunoy France; ^3^ School of Environmental and Geographical Sciences The University of Nottingham Malaysia Campus Semenyih Kajang Selangor Malaysia; ^4^ Centre for Biological Sciences Faculty of Natural and Environmental Sciences University of Southampton Southampton UK; ^5^ Kenyir Research Institute Universiti Malaysia Terengganu Kuala Nerus Terengganu Malaysia; ^6^ Department of Biological Sciences Sunway University Bandar Sunway Selangor Malaysia; ^7^ School of Natural Sciences and Engineering National Institute of Advanced Studies Bangalore India; ^8^ Department of Biology Faculty of Science Prince of Songkla University Hat Yai Songkhla Thailand; ^9^ Tree Climbers Malaysia Xtree Resources Shah Alam Selangor Malaysia

**Keywords:** antagonism, chiropterophily, ecosystem services, feeding behavior, fruit bat, mutualism, nectar robbing, network interactions, niche partitioning, pollen robbing, Pteropodidae

## Abstract

Fruit bats provide valuable pollination services to humans through a unique coevolutionary relationship with chiropterophilous plants. However, chiropterophily in the Old World and the pollination roles of large bats, such as flying foxes (*Pteropus* spp., *Acerodon* spp., *Desmalopex* spp.), are still poorly understood and require further elucidation. Efforts to protect these bats have been hampered by a lack of basic quantitative information on their role as ecosystem service providers. Here, we investigate the role of the locally endangered island flying fox *Pteropus hypomelanus* in the pollination ecology of durian (*Durio zibethinus*), an economically important crop in Southeast Asia. On Tioman Island, Peninsular Malaysia, we deployed 19 stations of paired infrared camera and video traps across varying heights at four individual flowering trees in a durian orchard. We detected at least nine species of animal visitors, but only bats had mutualistic interactions with durian flowers. There was a clear vertical stratification in the feeding niches of flying foxes and nectar bats, with flying foxes feeding at greater heights in the trees. Flying foxes had a positive effect on mature fruit set and therefore serve as important pollinators for durian trees. As such, semi‐wild durian trees—particularly tall ones—may be dependent on flying foxes for enhancing reproductive success. Our study is the first to quantify the role of flying foxes in durian pollination, demonstrating that these giant fruit bats may have far more important ecological, evolutionary, and economic roles than previously thought. This has important implications and can aid efforts to promote flying fox conservation, especially in Southeast Asian countries.

## 1 INTRODUCTION

Plant‐visiting bats of the family Pteropodidae are found throughout the tropics and subtropics of the Old World (Marshall, 1983; Mickleburgh, Hutson, & Racey, 1992). Pteropodids comprise almost 200 species (Simmons, 2005) with primarily phytophagous diets that include fruits, flowers, leaves, and other plant parts (though some also eat insects; Scanlon, Petit, & Sternberg, 2013); while some pteropodids are generalists and feed on a combination of these food items, others are strictly nectar feeding (Fleming & Kress, 2011; Marshall, 1985). Coevolution has produced unique relationships between these bats and plants that result in bat–flower and bat–fruit syndromes (Fleming, Geiselman, & Kress, 2009; Marshall, 1983), providing important ecosystem services through pollination and seed dispersal that benefit human well‐being either directly or indirectly (Fujita & Tuttle, 1991; Kunz, de Torrez, Bauer, Lobova, & Fleming, 2011; Scanlon, Petit, Tuiwawa, & Naikatini, 2014).

Flying foxes (*Pteropus* spp., *Acerodon* spp., *Desmalopex* spp.) are the largest pteropodids and the world's largest bats, with a geographical range that extends throughout the Old World from east Africa eastwards to the Pacific islands (Nowak, 1999). As a result of their large sizes, extensive foraging ranges, and mutualistic interactions with plants, they are considered to be necessary for maintaining the health of Palaeotropical forests, particularly on islands (Cox, Elmqvist, Pierson, & Rainey, 1991; Elmqvist, Cox, Rainey, & Pierson, 1992; Marshall, 1985; McConkey & Drake, 2006,2015; Scanlon et al., 2014). However, much of what is known on the specific ecological role of flying foxes has focused largely on seed dispersal (e.g., Deshpande & Kelkar, 2015; McConkey & Drake, 2006; Nakamoto, Kinjo, & Izawa, 2009; Nyhagen, Turnbull, Olesen, & Jones, 2005; Oleksy, Racey, & Jones, 2015; Richards, 1990). In Southeast Asia, investigations into bat pollination have typically focused on the smaller, nectarivorous (Stewart, Makowsky, & Dudash, 2014) pteropodids, showing how the maintenance of economically important fruit crops in the region rests upon the coevolutionary nature of bat–plant relationships (e.g., Acharya, Racey, Sotthibandhu, & Bumrungsri, 2015; Bumrungsri, Sripaoraya, Chongsiri, Sridith, & Racey, 2009; Bumrungsri et al., 2008; Srithongchuay, Bumrungsri, & Sripao‐raya, 2008). Such studies have yet to examine specific roles of the frugi‐nectarivorous (Stewart et al., 2014) flying foxes in chiropterophily—an aspect which remains poorly understood.

Chiropterophilous plants typically display bat–flower syndrome, that is, floral characteristics that are specifically adapted to attract large, nocturnal pollinators through visual and olfactory cues (Marshall, 1983). One particularly notable example of this is Southeast Asia's durian (*Durio zibethinus*), an important fruit crop throughout the region both culturally and economically (Start & Marshall, 1976). Although many modern agricultural cultivars are now popular, semi‐wild durian has long been grown for household consumption in Malaysia, Indonesia, and southern Thailand (Bumrungsri et al., 2009). In Malaysia, where the species is thought to be native (Morton, 1987; Subhadrabandhu & Ketsa, 2001), exports of durian fruits keep increasing annually and now contribute more than USD 17.9 million to the national economy (United Nations, 2016). This is probably just a tiny fraction of the economic value of the domestic trade within the country, where the popular fruit has high cultural importance and is considered the “king of fruits.”

Flying foxes and other pteropodids such as the cave nectar bat (*Eonycteris spelaea*) visit flowering durian orchards to feed (Aziz et al., 2017a; Bumrungsri et al., 2013; Gould, 1977, 1978; Soepadmo & Eow, 1976), leading to a perception among farmers that bats cause damage and negatively affect fruit production (Aziz, Olival, Bumrungsri, Richards, & Racey, 2016; Bumrungsri et al., 2009). Recent pollination experiments have shown that rather than being destructive, the cave nectar bat is actually a major pollinator for semi‐wild durian in southern Thailand (Acharya et al., 2015; Bumrungsri et al., 2009)—an example of chiropterophilous pollination syndrome playing an important role in both culture and economy. However, the role of larger fruit bats such as flying foxes in pollination ecology is still poorly understood and requires further elucidation. Early literature postulated that flying foxes likely have a negative impact in durian orchards, because they were believed to consume the whole flower or to destroy it through chewing (Lee, Norsham Suhaina, Boon, & Chua, 2002; Soepadmo & Eow, 1976; Start, 1974). However, these claims were not based on actual observations or empirical studies. In contrast, Gould (1977, 1978) reported, based on direct observations in an orchard, that *P. vampyrus* feeding in flowering durian trees only licked nectar from flowers and did not destroy or damage the flowers. Until now, however, no attempt has been made to test the role of flying foxes in durian reproductive ecology.

While exclusion experiments have been a successful approach for studying the role of smaller pteropodids in pollination (Bumrungsri et al., 2008, 2009; Srithongchuay et al., 2008), the comparatively greater sizes and different feeding behavior of flying foxes (Nathan, Karuppudurai, Raghuram, & Marimuthu, 2009; Nathan, Raghuram, Elangovan, Karuppudurai, & Marimuthu, 2005) are difficult to account for when using such a study design; it is challenging to design a specific treatment that can exclude flying foxes while still allowing access to smaller pteropodids. Consequently, exclusion experiments that included both flying foxes and smaller pteropodids have simply grouped them together as “bats” (e.g., Nathan et al., 2005, 2009).

Here, we use camera traps as a novel approach to investigate the role of the island flying fox (*Pteropus hypomelanus*) in the pollination ecology of durian trees on Tioman Island, Peninsular Malaysia, and to ascertain whether flying foxes have an effect on durian reproduction. Specifically, we asked the following questions related to the nature of the relationship between flying foxes and durian: (1) What animals visit durian flowers? (2) How do these animals interact with durian flowers over time and space? (3) How do bat–flower interactions affect pollination and reproductive success?

As flying foxes are still frequently hunted, persecuted and even legally killed as pests (Epstein et al., 2009; Fujita, 1988; Mildenstein, Tanshi, & Racey, 2016), such crucial information can improve our understanding of how their declines may impact the survival of chiropterophilous plants in the Palaeotropics. This will also help us better understand the roles of flying foxes as ecosystem service providers, which can then be used to justify their protection and conservation (Scanlon et al., 2014; Vincenot, Florens, & Kingston, 2017).

## 2 METHODS

This study conforms to the research ethics criteria stipulated by The University of Nottingham Malaysia Campus.

### 2.1 Study site

**Figure 1:**
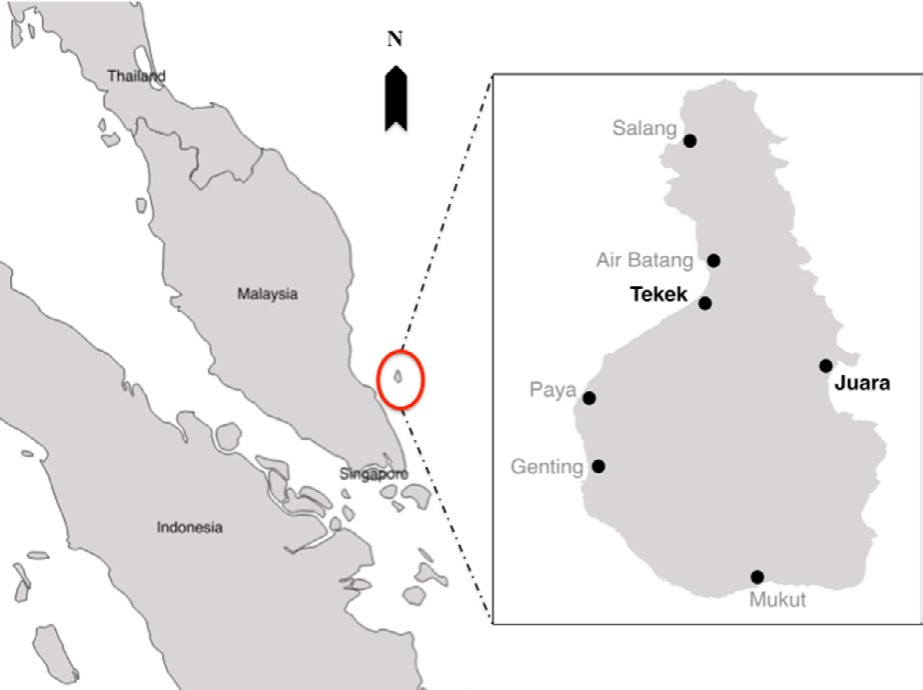
Map of Tioman Island, Peninsular Malaysia, and two villages where the island flying fox (*Pteropus hypomelanus*) can be found permanently roosting

All field data were collected on Tioman Island (2°48′38″ N, 104°10′38″ E; 136 km^2^; Figure 1), located 32 km off the east coast of Peninsular Malaysia in the State of Pahang, where permanent colonies of *P. hypomelanus lepidus* roost in villages and forage throughout the island (Bullock & Medway, 1966; Ong, 2000). Only four other pteropodid species have been recorded on the island—the lesser dog‐faced fruit bat *Cynopterus brachyotis brachyotis,* Horsfield's fruit bat *Cynopterus horsfieldi*, the cave nectar bat (Lim, Lim, & Yong, 1999), and dusky fruit bat *Penthetor lucasi* (Yong, Nawayai, Tan, & Belabut, 2012).

Much of the island inland is still covered by tropical rainforest, which has been designated as Pulau Tioman Wildlife Reserve (82.96 km^2^). It has a hilly topography, with flat areas only along the coast (Abdul, 1999). The area designated as a wildlife reserve is composed of lowland mixed dipterocarp forest and hill dipterocarp forest. Most forested areas are still inaccessible because of the rugged topography, with many steep slopes and rocky outcrops (Latiff et al., 1999). The climate is tropical, uniformly warm, and humid throughout the year (Hasan Basyri et al., 2001), but the northeast monsoon takes place from November to March (Bullock & Medway, 1966).

There are currently seven villages (Air Batang, Genting, Juara, Mukut, Paya, Salang, and Tekek) on the island, situated along the coastline (Figure 1). The majority of the local people are Muslim, and therefore, due to religious dietary restrictions do not hunt the bats for food or medicine. As the island's marine area is also a designated Marine Park and a popular tourist destination, many local people are involved in the tourism industry (Abdul, 1999). Our study was conducted during 21 April–29 July 2015 in one durian orchard in Juara, located next to the main road in the village. The durian trees ranged in height from 10 to 25 m; the exact ages of the trees were unknown, but the largest and tallest tree (D3) was said by the orchard owner to be around 90 years old. Only four durian trees in the orchard were flowering during the time of the study, while another four did not flower and could not be included in the study. The orchard generally had plenty of open space with no other cultivated fruit trees.

### 2.2 Study species

#### 2.2.1 *Pteropus hypomelanus*

**Figure 2:**
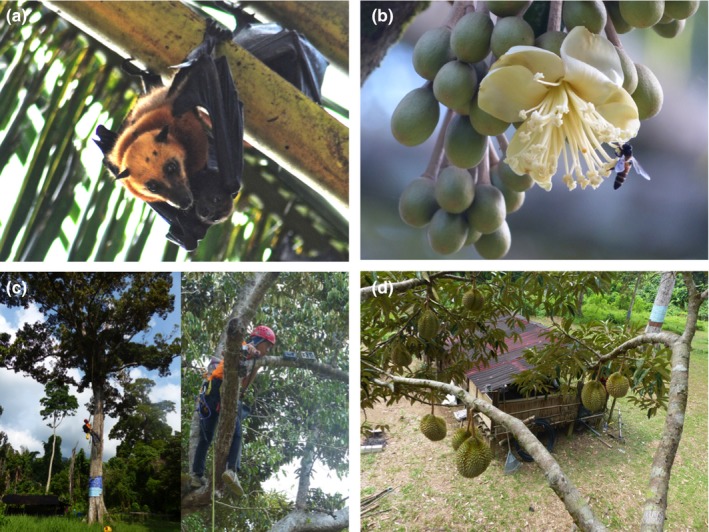
(a) Island flying fox (*Pteropus hypomelanus*); (b) Close‐up of durian flower showing *Apis dorsata* foraging on anthers; (c) Deployment of camera stations in durian (*Durio zibethinus*) trees; (d) Durian fruit set

The island flying fox (Figure 2a) is also known as the variable flying fox and, less commonly, as the small flying fox (Francis et al., 2008). It has a wingspan of more than 1 m and exhibits sexual size dimorphism, with males weighing around 570 g, and females around 470 g (Ouillette, 2006). It roosts gregariously, forming colonies of up to 5,000 individuals. It is a widespread insular species and considered to be abundant throughout its range, which extends from the Maldives and Indian islands in the west to Melanesia in the east. Because of this, it is considered to be Least Concern on a global scale by the IUCN Red List; however, its population trend is noted to be decreasing (Francis et al., 2008; Olival, 2008).

In Malaysia, *P. hypomelanus* is confined to small offshore islands where the country's only other flying fox species, *P. vampyrus* (large or Malayan flying fox), is usually not present (Pulliam et al., 2011), suggesting a certain degree of niche partitioning. A study on *Pteropus* population genetics and phylogeography (Olival, 2008) has shown the east coast populations off Peninsular Malaysia to be a subspecies—*P. hypomelanus lepidus*—genetically distinct from the west coast populations of *P. hypomelanus robinsoni*. The species is listed as Endangered on the Malaysian Red List (DWNP,^2010^), reflecting its precarious situation in the country.

On Tioman, *P. hypomelanus* can be found roosting permanently in two villages (Figure 1): Tekek, the main and biggest village (~1,260 people), located on the west coast, and Juara, the second largest village (~350 people) and the only one located on the east coast. Monthly roost counts conducted during March–October 2015 yielded estimated ranges of 675–1,033 individuals in Juara, and 1,503–4,352 individuals for Tekek.

Following Kingston (2010) and the Southeast Asian Bat Conservation Research Unit (SEABCRU; http://www.seabcru.org/?portfolio=flying-foxes), the common term “flying fox” is used here to refer only to the genera *Pteropus* and *Acerodon*; recent taxonomic revisions, however, have revalidated the genus *Desmalopex* (Almeida, Giannini, Simmons, & Helgen, 2014), which can now also be considered under this term. Thus, within the context and description of this specific study, “flying fox” is used to refer to *P. hypomelanus*, whereas the nectarivorous *E. spelaea* is referred to as “nectar bat.”

#### 2.2.2 *Durio zibethinus*

Durian (Family Malvaceae, previously Bombacaceae) is a tree likely native to Borneo, Sumatra and Peninsular Malaysia (Morton, 1987; Subhadrabandhu & Ketsa, 2001). Semi‐wild durian grown from seed and exposed to little or no artificial management is commonly planted in rural areas of southern Thailand, Malaysia, and Indonesia. Its seed results from open pollination, and its genetic diversity is reflected in a variety of taste and aril characters (Bumrungsri et al., 2009). Its flowers (Figure 2b) display typical chiropterophilous traits: large, strong, and wide‐mouthed, whitish or creamy in color, cauliflorous brush inflorescence, nocturnal anthesis lasting only for one night, and emitting a strong and distinctive odor (Marshall, 1983; Yumoto, 2000). A few durian cultivars are planted on a commercial scale. Hand‐crossed pollination is sometimes carried out in such commercial plantations.

Durian in Juara and Tekek has three distinct flowering seasons: April‐May, July–August, and October–November. However, each individual tree only flowers once a year, and the trees in this study all flowered from late April to early May. Due to constraints on accessibility (e.g., height of inflorescences, the presence of aggressive honeybees), we were unable to ascertain the full floral biology of the study trees, although flowers were observed to open and secrete nectar around 16:15 hr, and flower corollas dropped naturally from the trees between 01:00 hr and 02:00 hr. It is reasonable to assume that they possess floral biology characteristics similar to those reported by Soepadmo and Eow (1976) for Peninsular Malaysia, and Bumrungsri et al. (2009) for southern Thailand, for example, anthesis between 16:00 hr and 20:00 hr, anther dehiscence between 19:30 hr and 20:00 hr, stigma receptivity around 20:00 hr, nectar secretion rate peaking around 19:00 hr, and sucrose concentration of nectar highest in the early evening.

### 2.3 Camera‐trapping

On 21 and 23 April and 6 and 7 May 2015, once the durian trees in the orchard started flowering, we deployed 19 stations of paired infrared camera (Reconyx HC500) and video traps (Bushnell Trophy Cam) at four individual flowering trees (Figure 2c). Camera stations were placed across a vertical gradient between 2.4 and 20.3 m following each individual tree's unique structure and accessibility, and aimed at durian inflorescences at a distance of within 2 m to document the animal visitors and their feeding behavior. All cameras were removed on 29 July 2015.

An interaction was defined as physical contact between an animal and an inflorescence, regardless of whether this involved feeding or not. Both camera and video traps were set to allow a one‐second interval in between captures. The duration of each video capture was set for 10 s. Consecutive captures that depicted the same continuous physical contact were pooled together as one interaction. Spatiotemporal patterns were visualized in density plots using packages *overlap*,* reshape2,* and *ggplot2* in R statistical environment 3.2.2 (R Development Core Team, 2015). All mammal visitors were identified to species. Wherever possible, insects were identified to groups such as moths, stingless bees, and to species in the case of the Asian giant honeybee (*Apis dorsata*; Figure 2b). The frequency and duration of interactions between visitors and flowers were quantified, and the feeding behavior of visitors at inflorescences was also noted.

### 2.4 Direct observations

Camera‐trapping was supplemented with direct observations in the orchard during flowering. Daytime observations were carried out with binoculars during 6–8 May 2015 to record diurnal animal visitors and their feeding behavior. Nighttime observations of animal visits were carried out on 7 May 2015. Due to low visibility, these observations were conducted systematically every 30 min for each flowering tree using a thermalscope (Pulsar Quantum HD38S), starting from 19:30 hr (sunset at 19:17 hr) until 02:00 hr when flower corollas had dropped.

### 2.5 Bat sampling at flowering trees

In order to confirm the identity of the smaller pteropodids, mist‐netting was conducted in the durian orchard on 6 May 2015. To maximize the likelihood of capturing bats that had already fed in the durian trees, we avoided mist‐netting earlier in the evening. One mist net (2.6 × 12 m) was set up across a flyway between two durian trees, at a height of ~6 m. The net was manned and monitored directly from 20:30 hr, when it was first put up, until midnight when it was taken down and was never left unattended. It was checked every 15 min. Captured bats were identified to species following Kingston, Lim, and Zubaid (2006).

### 2.6 Effects of bat–flower interactions on pollination and reproductive success

We investigated the effects of flying fox [FF] and nectar bat [NB] interactions with flowers on fruit set by constructing generalized linear mixed‐effect models (GLMMs) that included all possible subsets using a multimodel inference framework (Burnham & Anderson, 2003). We tested frequency [FFF; NBF] and duration [FFD; NBD] of bat–flower interactions as covariates influencing pollination success, measured as initial fruit set after 20 days, and reproductive success, measured as mature fruit set after 60 days (Figure 2d). Initial fruit set was estimated from camera‐ and video‐trap footage 20 days after the first bat–flower interaction, as the late‐acting self‐incompatibility of the *D. zibethinus* breeding system causes unfertilized fruit to be aborted within this period (Bumrungsri et al., 2009; Honsho, Yonemori, Somsri, Subhadrabandhu, & Sugiura, 2004). As Bos et al. (2007) recommended that mature fruit set should be used as the metric for the economic role of pollinators, fruit set was quantified 60 days after the first bat–flower interaction, following Bumrungsri et al. (2009).

We included durian tree characteristic [DUR] as an additional covariate to distinguish one taller (~25 m) and isolated durian tree (D3) from the other three shorter (~10 m) and spatially clumped durian trees (D1, D2, and D4). To account for possible nonindependence due to other individual tree characteristics that we cannot account for (e.g., genetic variation), we also allowed model intercepts to vary across a random effect (*TRE*). We used Poisson's (log‐link) GLMMs to model the continuous response variables (i.e., fruit set). Before running the GLMMs, we first assessed whether the covariates were correlated (coefficient values >|0.5|) in order to obtain more stable and interpretable parameter estimates. We used sample size corrected Akaike Information Criterion (AICc) to determine the best candidate model, Akaike weights (wAICc) to quantify the probability by which a given model is the best within the candidate models set, and the sum of Akaike weights (SW) to estimate relative variable importance (Burnham & Anderson, 2003; Giam & Olden, 2016). We calculated $R_{m}^{2}$ to quantify the variance in the response variable that is explained by fixed effects in each GLMM (Nakagawa & Schielzeth, 2013). GLMMs were analyzed using packages *lme4* and *MuMIn* in R statistical environment 3.2.2 (R Development Core Team, 2015).

## 3 RESULTS

### 3.1 Animal visitors to durian flowers

From a survey spanning 54 days, we obtained 2,733 10‐s video clips and 3,367 still photographs of animal visitors from 13 camera stations (data from six camera stations could not be used due to malfunctions or inappropriate positioning). The number of inflorescences included within a camera's range varied from two to 19 (mean = 8; mode = 5). Our camera traps revealed six vertebrate taxa visiting durian flowers (Table 1). Two pteropodid bat species were photocaptured: The larger pteropodid was identified to be *P. hypomelanus*, while the smaller was identified as *E. spelaea*, based on Lim et al. (1999) and Yong et al. (2012). Invertebrate taxa, mainly *A. dorsata* and moths (Lepidoptera), were also photocaptured along with vertebrates in video clips, but only photocaptured independently in still photographs. Insect visitors could thus be quantified only from still photos, which were also used for quantifying flower abundance and fruit set; videos were more effective overall at identifying animal visitors, quantifying vertebrate–flower interactions, and observing feeding behavior.

**Table 1:** Durian tree characteristics and six vertebrate–flower interactions from camera traps at four durian trees in Juara, Tioman, 6 May – 29 July 2015

	Durian Tree D1	Durian Tree D2	Durian Tree D3	Durian Tree D4	Total	Mean	*SD*
Durian tree characteristics within detection range of camera traps							
No. of inflorescences	50	13	15	7	85	22	15
No. of estimated flowers	1,139	357	271	192	1,959	490	438
Fruit set at 10 days	0	0	0	0	0	0	0
Fruit set at 20 days	0	26	67	5	60	15	15
Fruit set at 30 days	0	19	28	0	48	12	15
Fruit set at 60 days	0	7	23	0	28	7	11
Island flying fox (*Pteropus hypomelanus*)							
Total no. of flower interactions	28	22	17	57	124	31	18
Duration (sec) of flower interactions	546	287	148	906	1,887	472	333
Mean duration (sec) of flower interaction	20	13	9	16	15	15	5
Nectar bat (*Eonycteris spelaea*)							
Total no. of flower interactions	759	11	5	367	1,142	286	358
Duration (sec) of flower interactions	1,821	24	12	863	2,720	680	859
Mean duration (sec) of flower interaction	2	2	2	2	2	2	0
Plantain squirrel (*Callosciurus notatus*)							
Total no. of flower interactions	23	18	0	71	112	28	37^a^
Duration (sec) of flower interactions	141	92	0	489	722	181	241^a^
Mean duration (sec) of flower interaction	6	5	0	7	6	6	1^a^
Long‐tailed macaque (*Macaca fascicularis*)							
No. of flower interactions	2	0	0	0	2	1	1
Duration (sec) of flower interactions	19	0	0	0	19	5	10
Mean duration (sec) of flower interaction	10	0	0	0	10	9	5
Colugo (*Galeopterus variegatus*)							
No. of flower interactions	0	0	0	1	1	0	1
Duration (sec) of flower interactions	0	0	0	11	11	3	6
Mean duration (sec) of flower interaction	0	0	0	0	0	0	0
Sunbird (Nectariniidae)							
No. of flower interactions	0	0	0	4	4	1	2
Duration (sec) of flower interactions	0	0	0	40	40	10	20
Mean duration (sec) of flower interaction	0	0	0	0	0	0	0
Insects^b^							
No. of flower interactions	521	612	15	6	1,154	289	323

a Calculations excluded D3 due to the absence of detections.

b Data from camera traps only; duration of flower interactions could not be inferred from photographs.

Bats were the most abundant overall of vertebrate visitors in the video‐trap footage, with *E. spelaea* being the most abundant species (Table 1). Based on the video footage, *E. spelaea* landed on inflorescences directly, head up and occasionally horizontally, thumb claws holding opened flowers, and inserting their muzzle into the corolla tubes of flowers. These visits lasted anywhere from less than a second to over one minute before the nectar bat would fly away again; the mean duration of visits was 2 s.

**Figure 3:**
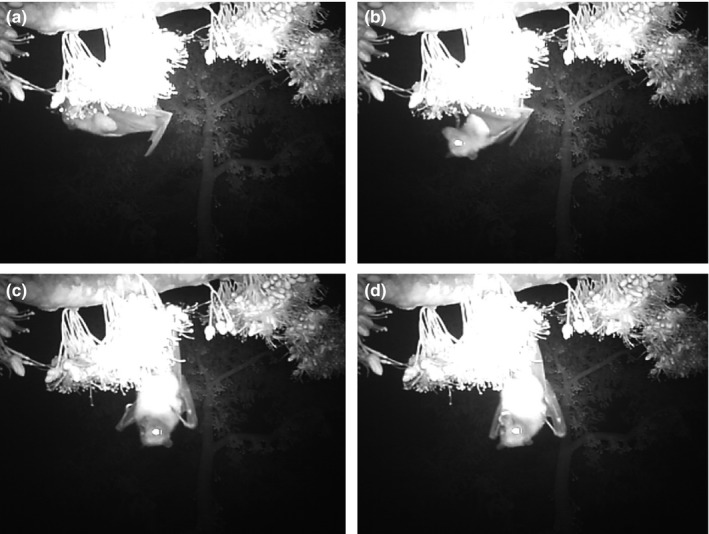
Screenshots of a video recording showing the island flying fox (*Pteropus hypomelanus*) feeding on durian nectar through a series of interactions: (a) insertion of mouth; (b) withdrawal of mouth; (c) resting on the branch; (d) licking of (presumably nectar) from mouth

In contrast, *P. hypomelanus* hung from the branch next to an inflorescence using their hind claws, occasionally using thumb claws to hold and move opened flowers closer toward them, and inserting their muzzle into the corolla tubes of flowers (Figure 3). Some *P. hypomelanus* occasionally hung from the inflorescence stalks using their hind claws while feeding. These feeding bouts could last for more than 1.5 min (mean duration 21 s). *P. hypomelanus* would either fly off after feeding or continue to hang from branches or inflorescence stalks even when not feeding, but would also move by crawling quadrupedally along a branch. Thus, *P. hypomelanus* was observed crawling from one branch to another, and also crawling from one inflorescence to another along the same branch to continue feeding.

**Figure 4:**
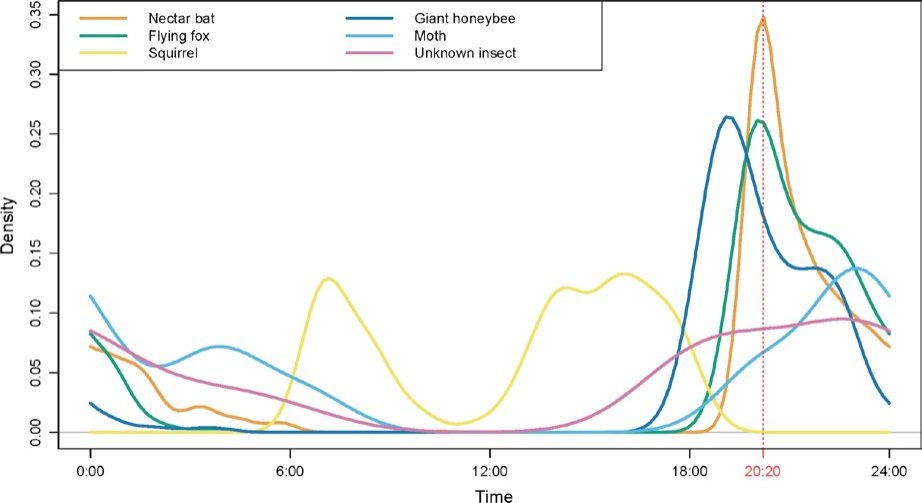
Daily temporal patterns of animal interactions with durian flowers between 6 and 20 May 2015. Nectar bat (*Eonycteris spelaea*), flying fox (*Pteropus hypomelanus*), and plantain squirrel (*Callosciurus notatus*) detections were amassed from 1,528 10‐s video clips. Giant honeybee (*Apis dorsata*), moth (Lepidoptera), and unknown insect detections were amassed from 948 camera‐trap photographs. Red dotted line represents peak activity (20:20 hr) for flying foxes

*Pteropus hypomelanus* and *E. spelaea* visited durian inflorescences in all flowering trees. *Pteropus hypomelanus* arrived first, around sunset (~19:20 hr), followed by *E. spelaea* at ~20:00 hr (Figure 4). *Pteropus hypomelanus* feeding activities were frequently accompanied by loud wing flapping and vocalizations throughout the night. *Pteropus hypomelanus* activity and noise decreased from 23:00 hr onwards, and by 00:15 hr no further calls were heard. However, on 7 May 2015 individuals were still directly observed roosting on branches in the durian trees even at 02:00 hr when observations ended.

Based on the video footage, the feeding behavior of *P. hypomelanus* on durian flowers was largely nondestructive, as they seemed to feed on nectar and did not consume the actual flowers. Flower damage observed from footage of feeding interactions (11 of 187 interactions) was minimal to nonexistent, similar to that caused by *E. spelaea* feeding (28 of 1,161 interactions). Thus, damage was restricted to the occasional loss of a few flower parts within an entire inflorescence and rarely whole flowers. Even physical movements by *P. hypomelanus* along tree branches only produced occasional, minimal loss of floral components. A single aggressive feeding interaction was recorded between two *P. hypomelanus* individuals, involving territorial behavior over the same inflorescence. All other feeding observations of *P. hypomelanus* were of individuals feeding solitarily on a branch. On five separate occasions *P. hypomelanus* individuals used a wing/forearm to push away *E. spelaea* from a nearby inflorescence. On two separate occasions, a *P. hypomelanus* was recorded repeatedly clapping its wings together rapidly, creating a loud noise.

The feeding behavior of plantain squirrels (*Callosciurus notatus*) was highly destructive. Forty‐two percent of *C. notatus* detections (Table 1) showed this squirrel species biting into the flowers directly, often at the base, tearing off pieces, and even whole flowers. We found that more than a quarter (27%, *n* = 669) of the dropped corollas found under two durian trees (D1 and D2; D3 was not in flower yet) were damaged, with holes in their bases (Table 2). The only tree without these damaged corollas on the ground was the tallest and oldest tree (D3), which was surrounded by open space. This tree was also the only one without any squirrel detections, probably due to its isolation from the rest of the trees. Squirrels were observed during the day in the other three trees, occasionally appearing to feed on durian flowers by nibbling at the bases.

**Table 2:** Damaged (i.e., holes in the bases) and undamaged (i.e., no holes) flower corollas found under durian trees

Tree	6 May 2015		7 May 2015		8 May 2015	
	Damaged	Undamaged	Damaged	Undamaged	Damaged	Undamaged
D1	13% (7)	87% (47)	13% (22)	87% (147)	0% (0)	100% (108)
D2	34% (25)	66% (49)	61% (103)	39% (65)	24% (23)	76% (73)
D3	–	–	0% (0)	100% (62)	0% (0)	100% (63)

Videos also showed that long‐tailed macaques (*Macaca fascicularis*) fed destructively, plucking flowers off with their hands and consuming these whole. However, *M. fascicularis* were only recorded feeding on two occasions. Sunbirds/spiderhunters (Family: Nectariniidae) were photocaptured feeding nondestructively during the day on four occasions, but before presumed full anthesis had occurred. One Sunda colugo (*Galeopterus variegatus*) was photocaptured during the study. Although it was briefly recorded brushing its face against flowers, the exact nature of the interaction could not be determined.

Video footage revealed that the most frequent vertebrate–flower interactions involved three mammal species—*E. spelaea* (83%), *P. hypomelanus* (9%), and *C. notatus* (8%)—over a period of 15 days. Based on 1,528 video clips, *P. hypomelanus* and *E. spelaea* were nocturnal and fed throughout the night, showing a slight temporal differentiation in feeding guilds (Figure 4). At dusk, *P. hypomelanus* generally arrived at the flowers first before *E. spelaea*, but the latter was usually the last to leave before dawn. Interaction occasions between *E. spelaea* and flowers surpassed those of *P. hypomelanus* around 19:00 hr but both peaked around 20:20 hr, which is close to the reported anthesis time (20:00 hr) for semi‐wild durian (Bumrungsri et al., 2009). The majority of bat visits took place within the effective pollination period for durian (~19:30–01:00 hr; Bumrungsri et al., 2009; Yumoto, 2000). *Callosciurus notatus* was diurnal, and its highest numbers of interactions with flowers were during mid‐morning and mid‐afternoon. There were insufficient detections of Nectariniidae, *M. fascicularis*, and *G. variegatus* to be used for quantifying temporal patterns.

**Figure 5:**
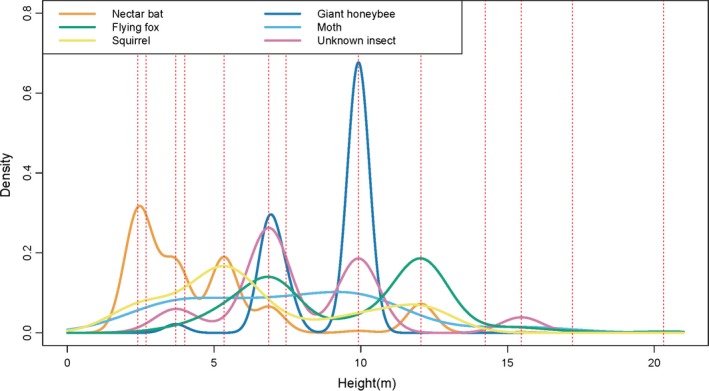
Spatial patterns of animal interactions between durian flowers along a vertical gradient between 6 and 20 May 2015. Nectar bat (*Eonycteris spelaea*), flying fox (*Pteropus hypomelanus*), and plantain squirrel (*Callosciurus notatus*) detections were amassed from 1,528 10‐s video clips. Giant honeybee (*Apis dorsata*), moth (Lepidoptera), and unknown insect detections were amassed from 948 camera‐trap photographs. Red dotted lines indicate heights at which cameras were deployed

Based on 1,146 videos of flower interactions involving *P. hypomelanus* and *E. spelaea* from 13 camera‐trap stations located along the vertical gradient (Figure 5), the number of *E. spelaea* interactions at inflorescences below 6 m was greater than that of *P. hypomelanus* interactions. Conversely, beyond this height, the amount of *P. hypomelanus* interactions consistently surpassed that of *E. spelaea* up to heights of around 20 m. We found, therefore, a clear vertical separation in feeding niches of these two bat species. *C. notatus* also fed at lower levels (≤5 m; Figure 5), whereas *A. dorsata* fed mostly in the middle (7–10 m; Figure 5).

Insect activity patterns could not be quantified accurately; as their small, fast, and ectothermic nature prevented accurate detection by the Bushnell video traps, insects could only be captured in video clips if there was a simultaneous detection of endothermic animals or wind movement. Reconyx camera traps successfully photocaptured insects independently of other animals, but only evening and nighttime data (using the infrared function) could be used, as daytime lighting prevented accurate identification of such small taxa. However, stingless bees (Family: Apidae) were directly observed feeding on durian flowers alongside *A. dorsata* shortly after the flowers opened in the late afternoon. Similarly to Bumrungsri et al. (2009) and Start (1974), we observed that the feeding behavior of the bees did not appear to facilitate pollination. Bees hovered around the ends of anthers, with stingless bees often forcing the anthers open and digging down into them before anthesis occurred, presumably to obtain pollen. Stingless bees never came into contact with stigmata, and giant honeybees only rarely, if ever.

### 3.2 Bat sampling at flowering trees

Four bats were captured during 3.5 net hours. Three were identified as *E. spelaea*, and one as *Cynopterus* sp. Pollen was found on the body of all *E. spelaea*, but none was found on the *Cynopterus* individual.

### 3.3 Animal–flower interactions and durian fruit set

Initial fruit set (pollination success) could be recorded from camera stations at three durian trees, but mature fruit set (reproductive success) could only be recorded at two durian trees, D2 and D3 (Table 1). Durian trees D2 and D3 that bore mature fruit had comparatively more *P. hypomelanus* interactions than *E. spelaea* interactions (27 vs. 12 and 18 vs. 3, respectively), while the two trees that did not bear mature fruit (D1 and D4) had comparatively more *E. spelaea* interactions than *P. hypomelanus* interactions (693 vs. 41 and 366 vs. 90, respectively).

D1 and D4 also had more *C. notatus* interactions than D2 and D3; although the two trees without fruit had the highest number of animal–flower interactions overall, including *P. hypomelanus* interactions, this also included the highest number of antagonistic interactions (e.g., removing or damaging flowers rather than pollinating them). In contrast, although the two trees with fruit had comparatively fewer *P. hypomelanus* interactions, they also had comparatively fewer antagonistic interactions overall.

### 3.4 Effects of bat–flower interactions on pollination and reproductive success

We obtained 1,419 video clips of bat–flower interactions at 86 inflorescences within the detection range of 13 camera stations distributed across four durian trees. In order to minimize collinearity (correlation coefficient values were <|0.5|) among covariates (FFI vs. NBI and FFD vs. NBD) and impacts of skewed data (i.e., camera stations had between two and 19 inflorescences within view) to achieve model convergence for the GLMMs, we only examined bat–flower interactions at the modal number of inflorescences (*n *= 5) per camera station. As such, only camera stations with at least five inflorescences were suitable (i.e., seven stations of 13), and only five inflorescences per station were included in GLMM analyses (35 inflorescences analyzed in total). These inflorescences were chosen based on their proximity to the camera to maximize detection probability. Also, when we included the number of flowers [FLO] per inflorescence in the global model, the top model showed that it did not have an effect on initial and mature fruit set, and was thus excluded to minimize model overfitting due to too many covariates.

We could not examine the effects of bat–flower interactions on pollination success, as the GLMMs failed to converge using the initial fruit dataset at 20 days (F20). However, we were able to examine the effects of bat–flower interactions on reproductive success (mature fruit set at 60 days; F60); GLMMs showed that the number of flying fox interactions [FFI], nectar bat interactions [NBI], and durian tree characteristic [DUR] appeared to influence mature fruit set [F60] (wAICc = 0.41, $R_{m}^{2}$ = 0.81; model 1 in Table 3a). In terms of relative variable importance assessed by sum of wAIC (SW), [DUR] was only slightly more important than [NBI] but relatively more important than [FFI] (SW = 0.79 vs. 0.70 vs. 0.55) in influencing mature fruit set [F60]. Durian tree characteristic [DUR] (older and taller) and [FFI] had a positive effect (conditional‐averaged coefficient = 0.44) on mature fruit set, but [NBI] had a slight negative effect (conditional‐averaged coefficient = −.11).

**Table 3:** The top three generalized linear mixed‐effect models (GLMM) showing the effect of (a) flying fox flower interactions [FFI], nectar bat–flower interactions [NBI] and durian tree characteristic [DUR]; and (b) duration of flying fox flower interactions [FFD], duration of nectar bat–flower interactions [NBD] and durian tree characteristic [DUR], on mature durian fruit set at 60 d [F60]. Each of four durian trees (*TRE*) was coded as a random effect

Model	*k*	LL	AIC_*c*_	dAIC_*c*_	*w*AIC_*c*_	$R_{m}^{2}$
(a)						
m1. F60 ~ FFI + NBI + DUR + (1|*TRE*)	5	−30	72	0.0	0.41	0.81
m2. F60 ~ NBI + DUR + (1| *TRE*)	4	−32	73	1.3	0.21	0.87
m3. F60 ~ DUR + (1| *TRE*)	3	−34	75	2.6	0.11	0.36
(b)						
m.1 F60 ~ NBD + DUR + (1| *TRE*)	4	−32	74	0.0	0.27	0.87
m.2 F60 ~ FFD + NBD + DUR + (1| *TRE*)	5	−31	74	0.1	0.25	0.80
m.3 F60 ~ DUR + (1| *TRE*)	3	−34	75	0.9	0.17	0.56

*k*, number of parameters; LL, maximum log‐likelihood; dAIC_c_, difference in AIC_c_ for each model from the most parsimonious model; wAIC_c_, AIC_c_ weight; $R_{m}^{2}$, marginal *R* ^2^ according to Nakagawa and Schielzeth (2013).

The relationship between the duration of bat–flower interactions and durian characteristic and mature fruit set [F60] were similar, albeit weaker (Table 3b). In terms of relative variable importance assessed by sum of wAIC (SW), [DUR] was again more important than [NBD] and [FFD] (SW = 0.74 vs. 0.60 vs. 0.38) in influencing mature fruit set [F60]; indeed, [FFD] is not important at all in this model. Durian tree characteristic, [DUR] (older and taller) and [FFD] had a very slight positive effect (conditional‐averaged coefficient = 0.02) on fruit set, while [NBD] had a slight negative effect (conditional‐averaged coefficient = −0.05).

## 4 DISCUSSION

This study has yielded important preliminary insights into the role of flying foxes in durian pollination. We obtained photographic and video evidence that *P. hypomelanus* feeds on the nectar produced by durian flowers. Camera‐trapping indicated vertical stratification in feeding niches among different bat species. Most importantly, we show that *P. hypomelanus* has a positive effect on durian reproductive success, suggesting a mutualistic relationship that developed through coevolution. However, our understanding of the strength of this relationship was compromised by the small sample size of trees and needs to be tested further by expanding the study into a more in‐depth and extensive investigation involving both isolated and nonisolated durian trees of varying heights from other orchard sites, and preferably over several durian seasons. The entire durian pollination network should ideally be studied at the community level (Memmott, 1999), taking into account mutualisms, antagonisms, and the dynamics of various inter‐species interactions within the pollination complex.

The evidence we have obtained disproves earlier assertions that flying foxes feed destructively on durian flowers (Soepadmo & Eow, 19761976; Lee et al., 2002; Start, 1974), and instead supports Gould's (1977, 1978) observations of nondestructive feeding. Such nondestructive feeding behavior has also been reported for flying foxes in kapok trees (*Ceiba pentandra*) in southern India (Nathan et al., 2005; Singaravelan & Marimuthu, 2004) and Madagascar (Andriafidison et al., 2006). Studies elsewhere corroborate our findings that demonstrate the potential of flying foxes to increase pollination success; for example, Elmqvist et al. (1992) showed through exclusion experiments that the kapok tree in Western Samoa depends entirely on flying foxes as pollinators. In subtropical Japan, flying foxes are the primary pollinators of the native plant *Mucuna macrocarpa* (Nakamoto et al., 2009; Toyama, Kobayashi, Denda, Nakamoto, & Izawa, 2012). Birt, Hall, and Smith (1997) examined pteropodid tongue ecomorphology and found that the structure of tongues and papillae of flying foxes support a role as pollinators. All these provide strong evidence that flying foxes are also important agents in chiropterophily.

### 4.1 Mutualistic and antagonistic network interactions in durian pollination ecology

Our study found the same type of animal groups as those reported visiting durian in southern Thailand (Bumrungsri et al., 2009) and kapok in southern India (Nathan et al., 2005). In addition, we discovered three further species of animal that also feed on durian flowers: squirrels (*C. notatus*), macaques (*M. fascicularis*), and a colugo (*G. variegatus*). The first two species fed destructively. The colugo has been reported before from durian orchards (Ketol, Abdullah, & Tedong, 2006), and although we could not ascertain its actual feeding behavior in this study, we may yet have obtained the first visual evidence showing that this animal uses durian trees as a food resource.

Like Bumrungsri et al. (2009) and Start (1974), we also found that a few sunbirds/spiderhunters (Nectariniidae) occasionally fed on durian flowers that had opened in the afternoon. However, as these interactions happened between 16:00 hr and 19:00 hr before full anthesis occurred, it was unlikely to result in pollination success (Soepadmo & Eow, 1976; Start & Marshall, 1976)—although this still needs to be verified. Stingless bees (Apidae) and *A. dorsata* also fed in the afternoon, with *A. dorsata* also feeding at night. Wayo and Bumrungsri (2017) have shown that bees can contribute slightly to pollination at least for the “Mon Thong” commercial cultivars in southern Thailand. However, our observations of bee feeding behavior at the semi‐wild durian trees in our study suggest that in this particular instance this animal group acted largely as pollen robbers, not pollinators. Moths, which feed only at night, could also play a role as pollinators, but in our study their impact was likely to be low as they were not camera‐trapped frequently. Also, Start (1974) observed moths feeding on durian nectar without actually coming into contact with either the anthers or stigma. Thus, in our study only the pteropodid bats seem to have a truly mutualist relationship with the durian tree.

The results of the GLMM analyses must be treated with caution. The inconclusive results for preliminary fruit set suggest an insufficient sample size and a need to replicate this study using more trees, more orchards, and more sites. However, the analysis for mature fruit set provides some useful clues for further investigation. For example, the taller and isolated characteristic of D3 tree appeared to have a positive effect on mature fruit set (Table 3). This positive effect could be due to fewer antagonistic interactions occurring at greater heights. In our study, fewer *A. dorsata* and *C. notatus* interactions occurred at the higher levels, where *P. hypomelanus* interactions were more numerous. Additionally, *C. notatus* was not detected in D3, possibly because it was surrounded by open space and the orchard owner had wrapped linoleum around the trunk to prevent access from the ground. The other trees had also received the same protective treatment; however, they were in close proximity to each other as well as other trees in the vicinity, essentially connected through a network of branches. *C. notatus* could thus easily cross over from one to tree to another using closely positioned branches as bridges. Damaged flower corollas, which were likely caused by *C. notatus* nibbling holes into the flower bases to access nectar, were not found under D3. Therefore, it is likely that this tree did not suffer any flower damage from *C. notatus*, which may be one reason why it produced the most fruits. In any case, we could not use GLMMs to determine whether *C. notatus* had any effect on fruit set because of zero‐inflated data (camera traps did not detect any *C. notatus* on D3). Durian produces copious numbers of flowers, and low levels of flower damage/loss may be negligible or even beneficial—too many flowers can lead to resource limitation, resulting in decreased fruit abundance and/or quality (Yumoto, 2000; S. Bumrungsri, unpublished). Thus, removal or nonpollination of excess flowers can actually improve fruit production. However, observations of *C. notatus* feeding behavior showed that their interactions with durian flowers were extremely destructive, and therefore, it is possible that a certain threshold number of individuals/visits could begin to have a detrimental effect on the tree's reproductive success. Trees in our study that did not produce fruits had more such antagonistic interactions than the trees that did produce fruit, and therefore, it is possible that taller and isolated trees may enjoy greater reproductive success; indeed, the potential importance of flying foxes for facilitating long‐distance pollen transfer among tall trees has already been observed in Australia (Bacles et al., 2009).

### 4.2 Implications of niche partitioning and feeding behavior on pollinator effectiveness

Clear temporal differentiation in visits has been observed elsewhere between flying foxes and other pteropodids feeding on flowering kapok in southern India (Nathan et al., 2005; Singaravelan & Marimuthu, 2004) and Madagascar (Andriafidison et al., 2006), involving differences of several hours. This temporal differentiation may be due to roost locations influencing commuting times and energetic requirements, but is likely also due to resource partitioning (Andriafidison et al., 2006; Nathan et al., 2009). In contrast, our study found only very slight temporal partitioning between flying foxes and nectar bats. Although *P. hypomelanus* arrived and left slightly earlier, the difference in timing was only 30–40 min, and peak activity for both bat species occurred at almost the same time. Nathan et al. (2009) found an even smaller time difference, of only ~15 min, between *P. giganteus* and *Cynopterus sphinx* arriving to feed on madhuca (*Madhuca latifolia*) flowers in southern India, but peak activity timings were 1 hr apart. In that study, however, flying foxes arrived later and left earlier. It is notable that the durian orchard in our study is located only ~300 m from the nearest *P. hypomelanus* roosts, which are situated on the beach in Juara, presumably allowing quick and early access by the larger pteropodid. Roost locations for *E. spelaea* were unknown, but the large cave roosts required by this nectarivorous species (Start & Marshall, 1976) were not observed anywhere in close proximity to the orchard. Nathan et al. (2009) have suggested that smaller bats may gain sufficient energy from a single flower, enabling longer commuting flights from more distant roosts (Start & Marshall, 1976). These factors may account for there being only a slight temporal difference between the two bat species.

Our study revealed a definite vertical stratification in the foraging heights of the two pteropodid species. Similar to findings from the above‐mentioned studies on kapok (Andriafidison et al., 2006; Nathan et al., 2005; Singaravelan & Marimuthu, 2004) and madhuca flowers (Nathan et al., 2009), flying foxes preferred the upper levels of a tree, whereas the smaller bats were more likely to feed in the lower levels. This vertical stratification was also observed for different pteropodid species feeding in fruit trees in southern India (Sudhakaran & Doss, 2012), and pteropodids caught in Fijian rainforest (Scanlon & Petit, 2016), and likely helps to avoid inter‐specific competition (Fischer, 1992; Fleming, 1979; Thomas & Fenton, 1978). Again, this spatial partitioning corroborates findings by Gould (1977, 1978), who reported that flying foxes visited most flowers in the upper canopy of durian trees, and that only small bats fed in the lower canopy. In our study, this spatial partitioning also appeared to influence the species of bat found feeding in durian trees of differing heights; comparatively more *P. hypomelanus* were found in D3, the tallest tree (~25 m), whereas comparatively more *E. spelaea* visited the shorter D1 (~10 m). This height differentiation suggests that semi‐wild durian, which tends to be taller than commercial cultivars, could be particularly dependent on flying foxes for reproductive success. As it is used as grafting and breeding stock for commercial cultivars, its continued survival is important for the commercial durian plantation industry.

Height differentiation may also possibly explain the surprising slight negative effect of *E. spelaea* interactions with durian flowers (Table 3). This negative effect appears to contradict all previous studies showing that this bat species is an effective and even principal pollinator promoting cross‐pollination of durian (Acharya et al., 2015; Bumrungsri et al., 2009). It is unclear why *E. spelaea* may have had a negative effect on reproductive success in our study; however, comparatively more mature fruit set was observed in the higher levels of trees, which coincides with higher *P. hypomelanus* interactions. In our study, fruit set was not correlated with flower abundance. Tree D1 had the highest number of flowers and highest overall number of *E. spelaea* interactions, yet no fruit set. This inverse relationship could be related to tree height and number of antagonistic interactions as mentioned above, but could also result from resource limitation (Yumoto, 2000), or the health and/or age of the tree. Pollination experiments have found that even hand‐crossed pollination conducted on 10‐year‐old durian around 10 m in height produced very few fruits; in addition, even older trees only set fruit in the higher branches (S. Bumrungsri, unpublished). If durian trees characteristically produce more fruits at greater heights, then *P. hypomelanus* may have served as a more important pollinator than *E. spelaea* in this particular study, because the former feeds in the higher levels of the trees.

It is also possible that perhaps *E. spelaea* feeding behavior simply does not transfer pollen as effectively as *P. hypomelanus*. Interestingly, Tschapka (2003) found that the perching behavior of frugivorous bats in Costa Rica feeding on flowers of the Neotropical palm *Calyptrogyne ghiesbreghtiana* facilitated better pollen transfer than the hovering behavior of nectarivorous bats. Although Sritongchuay and Bumrungsri (2016) have shown that nectarivorous bats have higher network strength than small frugi‐nectarivorous bats in mixed‐fruit orchards of southern Thailand, such differences in feeding behavior may also be a factor and should also be taken into account when assessing pollination effectiveness.

### 4.3 Implications of feeding behavior and pollinator dynamics on durian reproductive success

An alternative explanation for the apparent negative impact of *E. spelaea* on mature durian fruit set could be that excessive visits by pollinators might have a negative effect resulting in low fruit production. Such a scenario was observed by Wilmott and Búrquez (1996) for the self‐incompatible desert climbing vine *Merremia palmeri* of Mexico, where more than five visits by its primary pollinator actually resulted in lower fruit set. A similar effect has been postulated by Avila, Pinheiro, and Sazima (2015) for the generalist forest tree *Inga subnuda luschnathiana* in Brazil, where visitations by animals to flowers resulted in decreased fixed polyads in stigmas. If a similar scenario occurs for durian, *P. hypomelanus* may not have had this effect in our study since it fed solitarily, occupied the same branch for extended lengths of time even when not feeding, and due to its territorial feeding behavior would have defended its floral resources against other visitors. On the other hand, smaller bats, which do not defend feeding territories, may be more likely to congregate on flowers in larger numbers, resulting in more overall visits to individual flowers. It is thus possible that once the number of visits exceeds a certain threshold, pollination success may become less likely.

Differences in feeding behavior between pteropodid species may also be a factor influencing to what extent cross‐pollination occurs. Acharya et al. (2015) have shown how visits by nectar bats help to promote cross‐pollination by depositing conspecific pollen on stigmas. It would be interesting to investigate whether both inter‐specific and intra‐specific bat feeding interactions may exert a pressure that promotes cross‐pollination. McConkey and Drake (2006) have shown that high densities of flying foxes lead to more aggressive feeding interactions, which then facilitates more effective seed dispersal; such territorial feeding behavior by flying foxes has also been reported by Brooke (2001), Gould (1977, 1978), and Wiles, Engbring, and Falanruw (1991). Elmqvist et al. (1992) suggested that flying fox feeding behavior may also lead to density‐dependent pollen dispersal, transferring conspecific pollen both within and among trees, a theory also postulated by Gould (1977). In our study, only one aggressive feeding interaction was recorded between flying foxes. There were more incidents of flying foxes physically defending inflorescences against nectar bats than against other flying foxes; comparatively less aggressive feeding interactions took place between flying foxes, as almost all flying foxes fed solitarily without physical intra‐specific interference. This lack of interaction contrasts with Gould's (1977, 1978) observations of *P. vampyrus* feeding in durian trees, which involved many close agonistic encounters. However, given that our study recorded fewer flying foxes than expected given our population counts for Juara and the limited number of flowering durian trees during this period, it could be that alternative food sources were also available at the same time, thereby reducing the need for flying foxes to compete for durian flowers. Also, Gould (1977, 1978) observed flying foxes defending durian flowers from conspecifics using spread‐wing displays; this caused flying bats to veer away at a distance of 30 m without physical interactions taking place. Additionally, Brooke (2001), Trewhella, Rodriguez‐Clark, Davies, Reason, and Wray (2001), and Wiles and Conry (1990) all reported wing‐clapping similar to that which we observed, where wings were quickly brought together so that the forearms hit and created loud claps in order to threaten conspecifics during feeding. In our study, these spread‐wing and wing‐clapping threat displays, along with frequent vocalizations, may have been sufficient to deter conspecific competitors; Trewhella et al. (2001) note that only when such behavior was insufficient did more physical interactions take place.

Although we observed some loss of floral components in our study, the amount was small relative to the number of flowers in an inflorescence. This loss seemed far less substantial than the significant destruction caused by flying foxes to flowers and immature fruits of kapok in Samoa, where the tree appears to be completely dependent on a dystrophic pollination system in which its sole pollinator also destroys up to 50% of its flowers and fruit (Elmqvist et al., 1992). However, flying foxes in Madagascar were observed to feed on kapok nectar without causing significant damage (Andriafidison et al., 2006). In our study, bats never ate the durian flowers or fruits. The large numbers of flowers produced by durian probably means that the overall pollination benefits from flying fox visits outweigh the low amounts of occasional flower loss—an observation also made by Gould (1977). Similar observations were made by Nakamoto et al. (2009) and Toyama et al. (2012) for *M. macrocarpa* in Japan, noting that a certain amount of flower damage does not preclude flying foxes from effectively pollinating the plant.

Ultimately, this study provides valuable clues regarding the implications of flying fox declines throughout their range. It is possible that coevolution has produced interrelated population dynamics between “apex pollinators” (e.g., flying foxes) and “mesopollinators” (e.g., nectar bats), resulting in an effect similar to that of mesopredator release (Crooks & Soulé, 1999). In this case, “mesopollinator” visits, when no longer suppressed by the presence of a larger pollinator, may increase, leading to reduced pollination success, and negatively affecting a plant's reproductive ability. Cox and Elmqvist (2000) also proposed a similar “keystone pollinator” role for Samoan flying foxes that potentially shape ecosystem structures in a manner analogous to that of predators. Boulter, Kitching, Howlett, and Goodall (2005) suggested that plants with a generalist pollination system such as *Syzygium sayeri* can compensate for the absence of certain pollinators with the presence of other pollinators, in which case, the loss of a particular pollinating animal may not represent a significant difference. Plant species such as durian, however, clearly rely on a highly specialized pollination system that depends entirely on the nocturnal pteropodids that the plant has coevolved with over millions of years (Marshall, 1983). In this kind of system, the loss of an “apex pollinator” could well have reproductive consequences, even when another pollinator is still available to do the job.

### 4.4 Conservation implications

Given the potential importance of flying foxes in ensuring the continued reproductive success of durian trees, the economic implications for the durian fruit industry should not be ignored. The conservation value of such an economic role is obvious. It is particularly significant given that some commercial durian farmers, such as in southern Thailand, have resorted to artificial cross‐pollination by hand in the absence of natural pollinators—a laborious, time‐consuming, costly, and dangerous method (Wayo & Bumrungsri, 2017). On Tioman, local people hold negative perceptions and misconceptions of *P. hypomelanus* in addition to low awareness of bat ecosystem services, which can be an obstacle to conservation (Aziz, Clements, Giam, Forget, & Campos‐Arceiz, 2017b). Showing how *P. hypomelanus* is in fact an important durian pollinator provides yet another example of a bat‐serviced plant that has high value to humans—such case studies of ecosystem services can be used to develop a tailored approach to promote bat conservation among local communities, and overcome negative attitudes toward flying foxes (Scanlon et al., 2014).

## 5 CONCLUSION

This study is the first attempt to assess the specific role of flying foxes in durian pollination ecology. We have demonstrated that this can be successfully conducted using camera traps that provide both photographs and video footage—a novel approach for studying the ecological function of flying foxes, and which should be combined with exclusion experiments where possible. The results of our study have shown that flying foxes do visit flowering durian orchards to feed on nectar—and importantly, without causing damage to flowers. We also show that there is a greater density of flying foxes feeding at higher levels in the trees, which allows the use of camera‐trap data to determine the effect of flying foxes on reproductive success. Pollination by flying foxes appears to have a positive effect on mature fruit set—this expands the current scant body of knowledge on chiropterophily in the Old World. Due to small sample size, however, caution must be exercised in interpreting the data, and further replication with larger datasets is needed in order to test the validity of the results in this study. However, the ecological, evolutionary, economic, and cultural importance of this bat species should not be underestimated and warrants further exploration.

## AUTHOR CONTRIBUTIONS

Sheema Abdul Aziz conceived and designed the study, collected the data, analyzed the data, contributed equipment/materials/analysis tools, wrote the manuscript, prepared figures and/or tables, and reviewed drafts of the manuscript. Sara Bumrungsri conceived and designed the study, provided technical input and advice, and reviewed drafts of the manuscript. Gopalasamy Reuben Clements collected the data, analyzed the data, contributed equipment/materials/analysis tools, wrote the manuscript, prepared figures and/or tables, and reviewed drafts of the manuscript. Kim R McConkey provided technical input, collected the data, and reviewed drafts of the manuscript. Tuanjit Sritongchuay provided training, advice and input on study design, and reviewed drafts of the manuscript. Saifful Pathil and Muhammad Nur Hafizi Abu Yazid contributed equipment/materials and collected the data. Ahimsa Campos‐Arceiz contributed equipment/materials/analysis tools, provided input on data analysis, and reviewed drafts of the manuscript. Pierre‐Michel Forget provided input on study design, contributed equipment, and reviewed drafts of the manuscript.

## CONFLICT OF INTEREST

None declared.

## Supporting information

Video S1: Island flying fox (*Pteropus hypomelanus*) feeding on durian nectar.

Video S2: Cave nectar bats (*Eonycteris spelaea*) feeding on durian nectar.

Video S3: *P. hypomelanus* defending durian flowers from *E. spelaea*.

Video S4: Aggressive feeding interactions between two *P. hypomelanus* individuals.

Video S5: Territorial wing‐clapping behaviour by P. hypomelanus in response to *E spelaea*.
